# 1075. The MET Project: Distinguishing Multisystem Inflammatory Syndrome in Children from Typhus Using Artificial Intelligence

**DOI:** 10.1093/ofid/ofac492.916

**Published:** 2022-12-15

**Authors:** Angela Chun, Abraham Bautista, Chad Weatherly, Isabella Osuna, Kristiana Nasto, Flor M Munoz, Gordon Schutze, Sridevi Devaraj, Eyal Muscal, Kristen Sexson Tejtel, Tiphanie Vogel, Ioannis Kakadiaris

**Affiliations:** Baylor COM, Houston, Texas; University of Houston, Houston, Texas; University of Houston, Houston, Texas; Baylor COM, Houston, Texas; Baylor COM, Houston, Texas; Baylor College of Medicine, Houston, Texas; Baylor COM, Houston, Texas; Baylor COM, Houston, Texas; Baylor COM, Houston, Texas; Baylor COM, Houston, Texas; Baylor COM, Houston, Texas; University of Houston, Houston, Texas

## Abstract

**Background:**

Multisystem inflammatory syndrome in children (MIS-C) following SARS-CoV-2 infection shares features with other inflammatory states, notably Kawasaki Disease. The rickettsial infection murine typhus is also in the differential for MIS-C in endemic areas. As the therapeutic approaches differ, it is essential to distinguish these disorders soon after presentation, well before confirmatory serologic testing results. Our objective was to develop an algorithm to accurately predict MIS-C versus typhus.

**Methods:**

Retrospective review extracted demographic, clinical, and laboratory features available within 6 hours of presentation for 133 MIS-C and 87 typhus patients. 33 features were broken into 44 inputs and passed through an attention module to compute importance. Inputs were then entered into machine learning algorithms as MIS-C or typhus. Patients were divided into training and test cohorts respecting proportions in the dataset. An equation was built to calculate the “MET” (MIS-C versus endemic typhus) score.

**Results:**

MIS-C patients were younger (8.4 v 11.2 years, p< 0.0001) and the majority (71%) presented on day 4-6 of fever; most typhus patients (84%) presented with ≥6 days (mean 4.9 v 7.3 days, p< 0.0001). Typhus patients were more likely to have rash (86% v 51%, p< 0.0001) and MIS-C patients red eyes (71% v 36%, p< 0.0001), other features were similar. MIS-C patients had higher C-reactive protein levels (17.7 v 9.8 mg/dL), procalcitonin (14.0 v 0.48 ng/mL), fibrinogen (558 v 394 mg/dL) and neutrophil-to-lymphocyte ratio (12 v 3.5), all p< 0.0001, other parameters were similar. MIS-C patients were also more likely to have elevated troponin (0.48 v 0.01 ng/mL, p< 0.0001) and require intensive care (66% v 6%, p< 0.0001). A long short term memory network outperformed 6 other models (99% accuracy using all 33 elements). The MET score predicted MIS-C versus typhus with 90% accuracy using only 10 features (sensitivity 90%, specificity 90%).

**Conclusion:**

The clinical and laboratory similarities between typhus and MIS-C present challenges, but they can be reliably distinguished using artificial intelligence with as little as 10 features. Our ongoing interprofessional collaboration aims to make the MET score readily available to clinicians for use in patient encounters.

**Disclosures:**

**Flor M. Munoz, MD, MSc**, Gilead: Grant/Research Support|Moderna: DSMB|Pfizer: DSMB **Tiphanie Vogel, MD, PhD**, Moderna: Advisor/Consultant|Novartis: Advisor/Consultant|Pfizer: Advisor/Consultant.

